# Giant Meningioma Diagnosis and Clinical Treatment: A Case Report

**DOI:** 10.7759/cureus.67029

**Published:** 2024-08-16

**Authors:** Jose Valerio, Noe Santiago, Maria P Fernandez Gomez, Luis Rey Martinez, Andres M Alvarez-Pinzon

**Affiliations:** 1 Neurological Surgery, Palmetto General Hospital, Hialeah, USA; 2 Neurological Surgery, Larkin Community Hospital, Miami, USA; 3 Neurological Surgery, Latinoamerica Valerio Foundation, Weston, USA; 4 Pathology and Laboratory Medicine, Palmetto General Hospital, Hialeah, USA; 5 I-Health Institute, Florida Atlantic University (FAU) Charles E. Schmidt College of Medicine, Boca Raton, USA; 6 Cancer Neuroscience Program, Institute of Neuroscience of Castilla y León, University of Salamanca, Salamanca, ESP

**Keywords:** drop foot, lower motor neuron, upper motor neuron, simpson grading scale, giant meningioma

## Abstract

This case report shows the importance of semiology during a clinical examination not only to diagnose spine clinical symptoms but also to review the central nervous system tumor as a foot drop cause. We report a unique case of a patient who consulted for constant progressive numbness and motor symptoms in the right lower extremity. Lumbar CT and MRI were negative for acute or chronic lumbar pathology. This is a 41-year-old female patient with a history of eight-month progressive right leg weakness. The physical examination did not reveal neurological alterations besides foot drop. MRI and lumbar X-rays showed no significant findings. Electromyography (EMG) indicated right peroneal neuropathy. Based on these findings, surgical treatment was not indicated; therefore, physical therapy and a referral to neurology were indicated. However, symptoms increased, resulting in a right lower extremity hemiparesis. A brain MRI showed a left frontoparietal giant meningioma, which was surgically resected after embolization. The patient evolved with a full recovery of the right-sided hemiparesis after surgery. Our case highlights the foot drop's multiple etiologies. Initially, a lumbar disc hernia was suspected, but it was ruled out by imaging studies. Later, the EMG revealed peroneal neuropathy, leading to a neurology consult. Unexpectedly, a giant intracranial meningioma was found, a rare case of foot drop. A consideration of upper motor neuron (UMN) and lower motor neuron (LMN) syndromes aided diagnosis. Tumoral resection with embolization resulted in significant improvement, showcasing the complexities of such cases. Foot drop is a peculiar clinical manifestation that must have an integral assessment to rule out peripheral and central causes. Even rare, giant meningiomas can cause focalized symptoms such as foot drop. Therefore, the assessment of foot drop should include the CT and MRI of the central nervous system.

## Introduction

Meningioma accounts for 39% of all brain tumors; therefore, it is the most common primary intracranial tumor in neurosurgery-oncology. These kinds of tumors are more common in females, with an estimated sex ratio of 2:1 (female/male), and this supports that the development of meningiomas is influenced by hormones, mainly by estrogen and progesterone in males and females [1].

Giant meningiomas are defined as a diameter longer than 5 cm of the tumoral mass [2,3]. According to Özsoy et al., giant meningiomas accounted for 35% of all intracranial meningiomas in their institution [4]. These tumors can reach significant sizes, potentially causing mass effects, brain compression, and neurological deficits due to their expansive growth. Symptoms may include headaches, seizures, cognitive changes, and focal neurological impairments, depending on the tumor's location. Diagnosis is usually confirmed through imaging studies, such as MRI or CT scans. Treatment often involves surgical resection, which may be complex due to the tumor's size and proximity to critical brain structures [3,4].

The aim of this manuscript is to report a case of a patient who consulted for constant progressive numbness and motor symptoms in the lower extremity. Lumbar CT and MRI were negative for acute or chronic injuries.

## Case presentation

**Figure 1:**
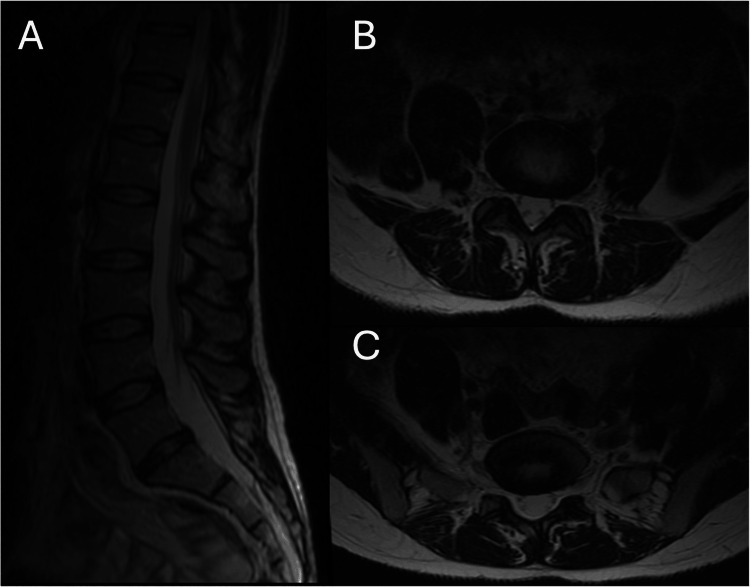
Lumbar MRI. Sagittal (A) and axial L4-L5 (B) and L5-S1 (C) MRI showed a bulging disc at L4-L5 and L5-S1 levels and no evidence of nerve root compression.

A 41-year-old female patient with a history of foot drop presented to our clinic due to moderate, progressively worsening right lower extremity weakness. Slurred speech, blurred vision, dizziness, fecal and urinary incontinence, and retention were denied. Her physical examination evidenced right lower limb strength 3/5, deep tendon reflexes 2+ in both lower extremities, intact sensation, negative straight leg raise test bilaterally, negative sacroiliac joint maneuvers bilaterally, and negative pain with the flexion and extension of the lumbar spine. The right foot drop was present, and there was no strength with the internal or external rotation of the foot. Likewise, the patient denied saddle anesthesia. A lumbar MRI was performed (Figure 1).

**Figure 2:**
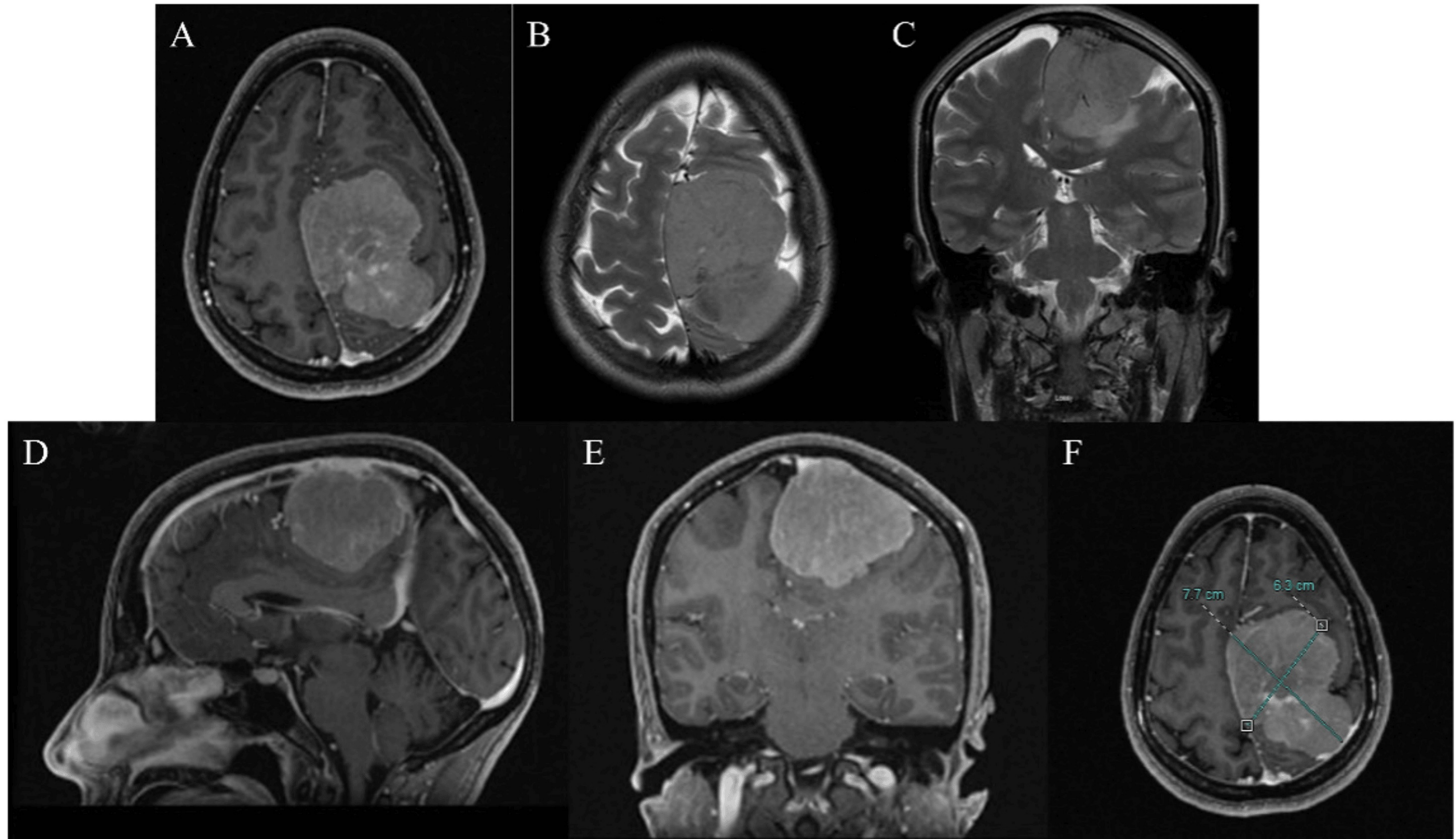
Brain MRI images show hyperintensity in the left frontoparietal lobe that enhances with contrast and with evidence of the pia mater on the T2 image and midline shift with mass effect with cingulum herniation and lateral ventricle compression at the level of the corpus callosum body and the splenium of the corpus callosum. (A) Axial MRI shows a giant meningioma that enhances with contrast with mass effect. (B) Axial T2 MRI shows an isointense meningioma with a partial arachnoid plane. (C) Coronal T2 MRI shows a left parasagittal meningioma with edema at the cortical level suggesting parenchymal infiltration and related to the affected motor area. (D) Sagittal T2 MRI shows parasagittal meningioma at the middle third of the superior longitudinal sinus related to the motor and sensitive areas. (E) The coronal view shows a parasagittal meningioma, which enhances with contrast. (F) The axial view shows the larger diameter of the tumoral mass (7.7 × 6.3).

**Figure 3:**
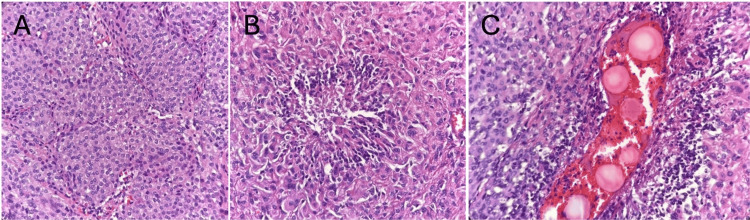
Histology. Meningothelial meningioma, WHO grade 1 out of 3, with lobular growth pattern and appearance (A); meningioma cells with a distinct focus on central necrosis, secondary to prior tumor embolization (B); and meningioma cells with a focus on necrosis and intravascular beads secondary to tumor embolization (C).

The electromyography (EMG) revealed moderate right axonal distal peroneal neuropathy and moderate denervation suggestive of peroneal nerve dysfunction. Left lower limb EMG was normal. At that moment, no neurosurgical intervention was recommended. Likewise, the patient was referred to physical therapy and neurology. However, symptoms never improved, and approximately one month later, the patient came to the emergency room since her right lower extremity symptoms worsened and progressed to grasping difficulty on her right upper extremity. A brain MRI was released, evidencing the presence of a parasagittal frontoparietal left giant intracranial tumor, with contrast enhancement, suggesting a meningioma (Figure 2). The tumoral mass had a diameter of 7.7 cm, causing superior longitudinal sinus displacement without compromising the vessel's lumen and only affecting the lateral wall. An angiography was performed to embolize the tumoral mass. Later, a complete tumoral resection was achieved; however, the superior longitudinal left sinus wall was coagulated, achieving a resection grade of Simpson II. Figure 3 shows the histological section.

**Figure 4:**
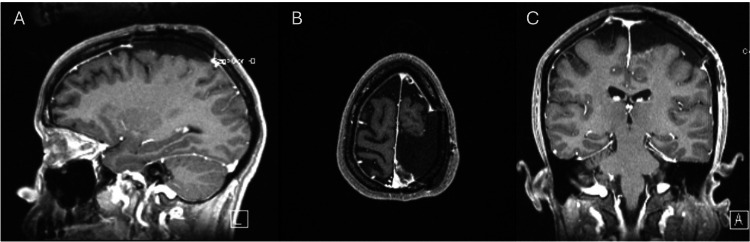
Postoperative MRI. (A) Sagittal MRI shows a gross total resection of the meningioma with a patent superior longitudinal sinus, (B) axial MRI does not show contrast-enhancing of residual tumor, and (C) coronal MRI shows a complete meningioma resection and the absence of cortical edema, which was observed prior to surgery.

The patient's outcome was favorable days after surgery; she reported significant improvement in her right-sided strength, as well as an improvement in her right foot drop. Figure 4 shows the postoperative control.

## Discussion

Meningiomas can be classified according to their size; large meningiomas have a diameter larger than 3 cm (about 1.18 in) [2], while meningiomas larger than 5-6 cm (about 1.97 in) are classified as giant [2,3,5,6]. Meningiomas with a diameter smaller than 2.5 cm (about 0.98 in) occasionally generate symptoms within five years of being discovered [7]. Meningiomas' clinical manifestations depend on their location and the mass effect of the tumor. However, some tumors grow over time without manifesting clinical symptoms and therefore debut with a considerable size. The reason why meningiomas can grow disproportionately causing no symptoms, regardless of their location within the brain, is unknown [3]. Common symptoms of meningiomas include headache, epilepsy, dementia, mental status changes, the loss of bladder control, and paresis [8,9]. According to the European Association of Neuro-Oncology (EANO) guidelines, location-specific symptoms include unilateral weakness, visual field loss, changes in personality, or speech problems [7]. Based on the location-specific symptoms described by the EANO guidelines, meningiomas can cause localized symptoms such as in our present case. Usually, due to their size and localization, giant intracranial meningiomas cause symptoms such as headaches, seizures, or hemicorporeal motor deficits. Infrequently, giant meningiomas do not cause focal deficit symptoms such as foot drop, thus thinking a giant meningioma causing a footdrop is rare [2,8]. Foot drop diagnosis might be challenging since it is difficult to differentiate between radiculopathy and peripheral nerve injury or neurological systemic disease [10].

To our knowledge, until 2021, only seven foot drop cases due to meningioma are reported in the literature [11]. Therefore, our case report would be the eighth case and is a parasagittal meningioma, which is defined as a meningioma that develops from at least one wall of the superior sagittal sinus. Its clinical manifestations are not so different from convexity meningiomas, which include headache, motor disturbances, and seizures [12]. However, our patient only coursed with foot drop, a motor disturbance, leading to a harder initial assessment.

This clinical scenario represents a challenge in its management due to rare clinical findings, diagnosis, and management. Foot drop is a common clinical sign that leads to several differential diagnoses, with lumbar disc hernia being the most frequent etiology and brain lesions the less frequent one [11,13,14]. Based on the differential diagnosis and epidemiology, our patient's assessment started with lumbar imaging studies; however, no lumbar etiology was found. The patient underwent electromyography, which evidenced moderate right-sided distal axonal peroneal neuropathy and moderate denervation, suggesting a peroneal nerve dysfunction. These findings led to a neurology consult for further assessment. The progress course of the patient and the lack of a diagnosis led to a central nervous system assessment. The finding of a left frontoparietal parasagittal giant meningioma in the imaging studies was an unusual finding, leading to a deep case analysis.

Giant meningioma as foot drop etiology constitutes a challenge, particularly when the initial spine assessment is negative [10]. Likewise, we consider that upper motor neuron (UMN) and lower motor neuron (LMN) syndromes should be considered to discard the brain from nerve causes. UMN syndrome is manifested as hyperreflexia, spasticity, and a positive Babinski reflex; on the other hand, LMN is manifested as hyporeflexia, flaccid paralysis, fasciculations, and atrophy [15]. As mentioned before, foot drop's most common cause is L4-L5 radiculopathy, which is usually caused by a herniated disc or a foraminal stenosis, leading to LMN clinical manifestations. However, when LMN causes have been ruled out, UMN causes should be elucidated [16]. Despite the clinical manifestations of UMN and LMN syndromes, our patient did not course with hyperreflexia, spasticity, or positive Babinski reflex, making our assessment harder.

Once foot drop etiology has been elucidated, subsequent management must be determined. In our case, our patient underwent a tumoral resection with previous embolization. Being the last one is an aid to decrease tumoral irrigation due to vessel occlusion [2]. After the tumoral resection of a giant meningioma, a Simpson II was achieved, meaning that the superior longitudinal sinus was not resected. The patient's symptoms improved, leading to a completely right-sided foot strength recovery after multidisciplinary management, as currently recommended [10]. Table 1 summarizes the Simpson grading system [17,18].

**Table 1:** Meningioma Simpson grade.

Grade	Definition	Reported recurrence
0	Additional 2 cm dural margin removal around the tumor	0%-1.8%
I	Complete macroscopic tumor removal + the removal of the affected dura and bone. Some cases might require venous sinus resection when a tumor arises from the dural venous sinus wall	9%
II	Complete macroscopic tumor removal + the coagulation of the affected dura	19%
III	Complete macroscopic tumor removal	29%
IV	Partial resection. In situ, the intradural tumor is left	39%
V	Decompression ± biopsy	88.9%

Giant meningioma increases the overall complications compared to meningiomas; this has been reported by Haeren et al. and corroborated with the literature, since their complication rates increased from 57% to 64% in patients with giant meningioma diagnosis. Likewise, they compared their rates with the literature, finding a rate range from 46% to 59% in giant meningioma complications [6]. Teama et al. did a study in which 48 patients with giant meningioma were included, from which new cranial nerve (CN) deficit occurred in 14 cases (29%), and only eight patients had complete recovery within three months with a return to normal status; however, the remaining six patients had a sustained cranial nerve deficit, including four patients who lose the olfaction, one case with moderate facial palsy, and one case with trigeminal nerve affection. Hemiparesis was described in three cases, and the cerebrospinal fluid leak was found in seven cases; nevertheless, this complication only required conservative management and stopped within one week in all cases [7]. Yin et al., in a 66-case study of patients diagnosed with giant meningioma, reported neurological complications such as hemiplegia, hemiparesis, CN VII palsy, visual defect, decreased hearing, aphasia, mental disorder, infection, and seizure. Hemiparesis was the major postoperative neurological deficit [19]. Despite all the described complications, our patient did not course with any of them; contrarily, our patient coursed with an improvement in her foot drop.

## Conclusions

Foot drop is a unique clinical manifestation that requires a thorough assessment to rule out both peripheral and central causes. Although uncommon, giant meningiomas can lead to focalized symptoms such as foot drops. Therefore, this unique case report finding recommends an imaging assessment that includes a CT scan and brain MRI to rule out tumoral brain causes. More research is needed to evaluate the appropriate state-of-the-art diagnosis for giant meningioma associated with foot drop.
